# On the value and limitations of incorporating a “clean phase” into the surgical treatment of prosthetic joint infections – an illustrative cadaveric study using fluorescent powder

**DOI:** 10.1186/s40634-022-00467-x

**Published:** 2022-03-21

**Authors:** Georges Vles, Jeroen Bossen, Johannes Kloos, Philippe Debeer, Stijn Ghijselings

**Affiliations:** 1 Department of Development and Regeneration, Faculty of Medicine, Institute for Orthopaedic Research and Training (IORT), Leuven, KU Belgium; 2 Division of Orthopaedics, University Hospitals Leuven, Leuven, Belgium

**Keywords:** Prosthetic joint infection, Clean phase, Fluorescent powder, Cadaveric study, Revision arthroplasty, Surgical technique, Contamination, Bacterial load reduction

## Abstract

**Purposes:**

A septic revision of an artificial joint is routinely split up in a so-called *dirty phase* and a *clean phase*. The measures taken to initiate the start of the clean phase vary significantly between musculoskeletal infection centers. We performed simulations of one-step exchanges of infected THAs and sought to 1) determine the effect of different clean phase protocols on the sterile field, and 2) determine whether or not it is possible to re-implant the new prosthesis completely clean.

**Methods:**

Nine fresh frozen cadaveric hips were used and primary THA was undertaken via a direct anterior approach. Before implantation of the components varying amounts of fluorescent powder (GloGerm) were deposited, simulating bacterial infection. Second, a one-step exchange was performed via a posterolateral approach. After implant removal, debridement, and lavage, randomization determined which clean phase protocol was followed, i.e. no, some or full additional measures. Finally, the new prosthesis was re-implanted.

In order to determine the effect of different clean phase protocols on contamination of the sterile field standardized UV light-enhanced photographs were obtained of 1) the gloves, 2) the instrument table, 3) the drapes, and 4) the wound and these were ranked on cleanliness by a blinded panel of hip surgeons.

In order to determine whether or not it is possible to re-implant the prosthesis completely clean, the implant was taken out again at the end of the one-step exchange and inspected for contamination under UV light.

**Results:**

The gloves, the instrument table, the drapes and the wound were significantly cleaner after a clean phase using full additional measures compared to partial or no additional measures (*p* < 0.000)*.* Partial measures were able to reduce some of the contamination of the gloves and the wound, but had no effect on the drapes and the instrument table. All re-implanted implants were contaminated with some amount of fluorescent powder at the end of the one-step exchange.

**Conclusions:**

We advise to incorporate a clean phase with full additional measures into the surgical treatment of prosthetic joint infections, as partial measures seem to be a poor compromise.

**Level of evidence:**

Not applicable (cadaveric study).

**Supplementary Information:**

The online version contains supplementary material available at 10.1186/s40634-022-00467-x.

## Introduction

Prosthetic Joint Infection (PJI) remains a devastating complication following an otherwise highly successful surgical intervention and generally requires re-operation [1–4]. Regardless whether the next procedure is a Debridement, Antibiotics and Implant Retention (DAIR), a one-step exchange or the 1st stage of a two-step exchange, there routinely is a *dirty phase* and a *clean phase* [5]*.* During the dirty phase the bacterial load is reduced as much as possible by debridement of infected and necrotic tissue and by extensive pulsatile lavage with saline ± antiseptics. At one point, one needs to re-implant the mobile parts, the entire implant or the spacer, depending on the chosen strategy, and this marks the beginning of the clean phase.

There is considerable variation in practice between well-established musculoskeletal infection centers regarding the steps just before the initiation of the clean phase. Some will take no additional measures, i.e. after debridement and lavage the clean phase begins, while others will close the wound, get rescrubbed, re-prep and re-drape the surgical field, and use new instruments to re-implant the necessary parts and finish the operation [6–9]. The latter is of course accompanied with extra time, effort and costs and there are no studies to support or negate its superiority. Therefore, most surgical teams will only take some additional measures, e.g. change gloves, the tip of the diathermy and suction, and place one extra drape [10, 11]. 

The question remains if we are not selling our patients and ourselves short by this compromise. We therefore performed simulations of one-step exchanges of infected total hip arthroplasties on cadavers in order to 1) determine the effect of different clean phase protocols on the sterile field, and 2) determine whether or not it is possible to re-implant the prosthesis completely clean.

## Materials and methods

### Study design and setting

**Fig. 1:**
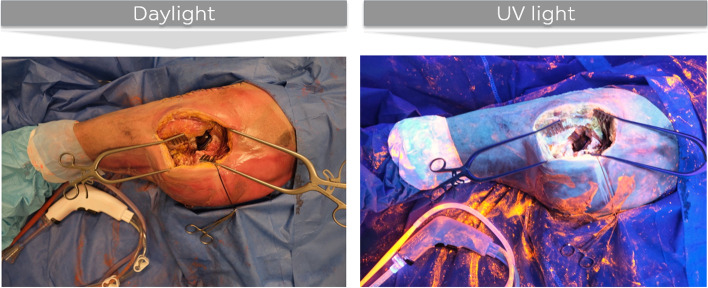
The appearance of orange fluorescent powder (GloGerm, Hygienic Solutions, Lincoln, UK) under normal daylight and under UV light. No additional measures protocol (bacterial load of 3 × 2,5 mg GloGerm powder)

This cadaveric study was approved by our ethical review board and registered at the Belgian National Council for Bioethics (NH019 2021–6-01). The implant investigated was an uncemented Total Hip Arthroplasty (THA, Pinnacle cup, Corail Stem, DePuy Synthes, Warsaw, Indiana) and bacterial load was simulated using a fluorescent powder (GloGerm, Hygienic Solutions, Lincoln, UK) frequently used in studies on contamination [12, 13]. These 5 µm particles are the same size as bacteria and appear orange under UV light (Fig. 1). All experiments were carried out at the Vesalius institute of the Catholic University of Leuven, Belgium.

### Cadaveric specimens

Nine fresh frozen cadaveric hips (5 left, 4 female, mean age 85 years SD 8.5, mean weight 73 kg SD 17) were used.

### Description of experiment

**Fig. 2:**
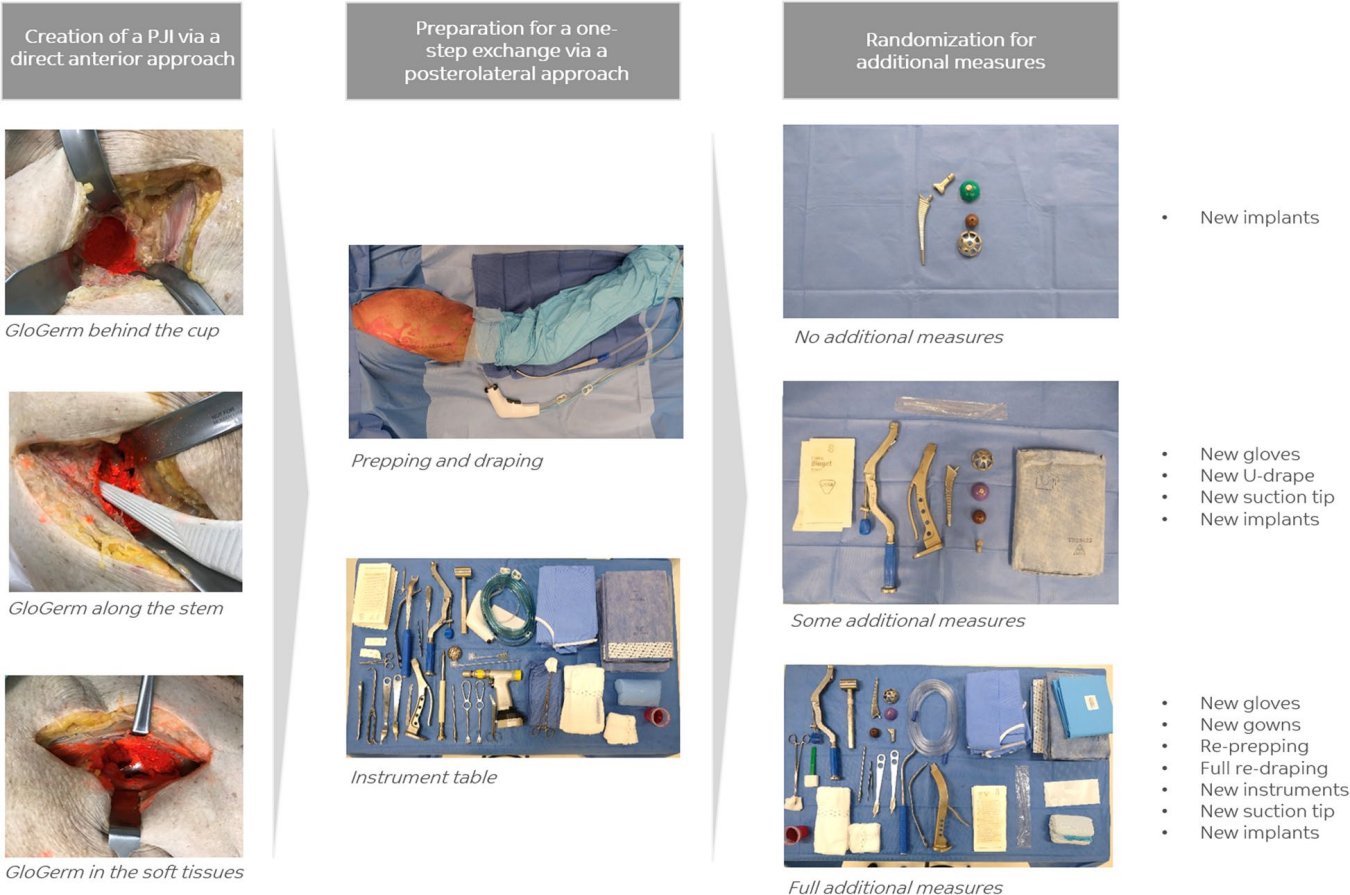
Simulating an infected Total Hip Arthroplasty

First, a PJI of a THA had to be simulated (Fig. 2). The cadaver was placed supine and a primary THA was undertaken via a direct anterior approach. Right before implantation of the components 1, 2.5, or 5 cc of orange fluorescent powder was placed in the already prepared acetabulum and an equal volume inside the femur. Next, the components were implanted, the joint was relocated and another 1, 2.5 or 5 cc of powder was distributed around the implant in the soft tissues. After full closure, the skin of the cadaveric specimen was checked for contamination with fluorescent powder using a UV light source. If this was the case, the specimen was washed until clean.

**Fig. 3:**
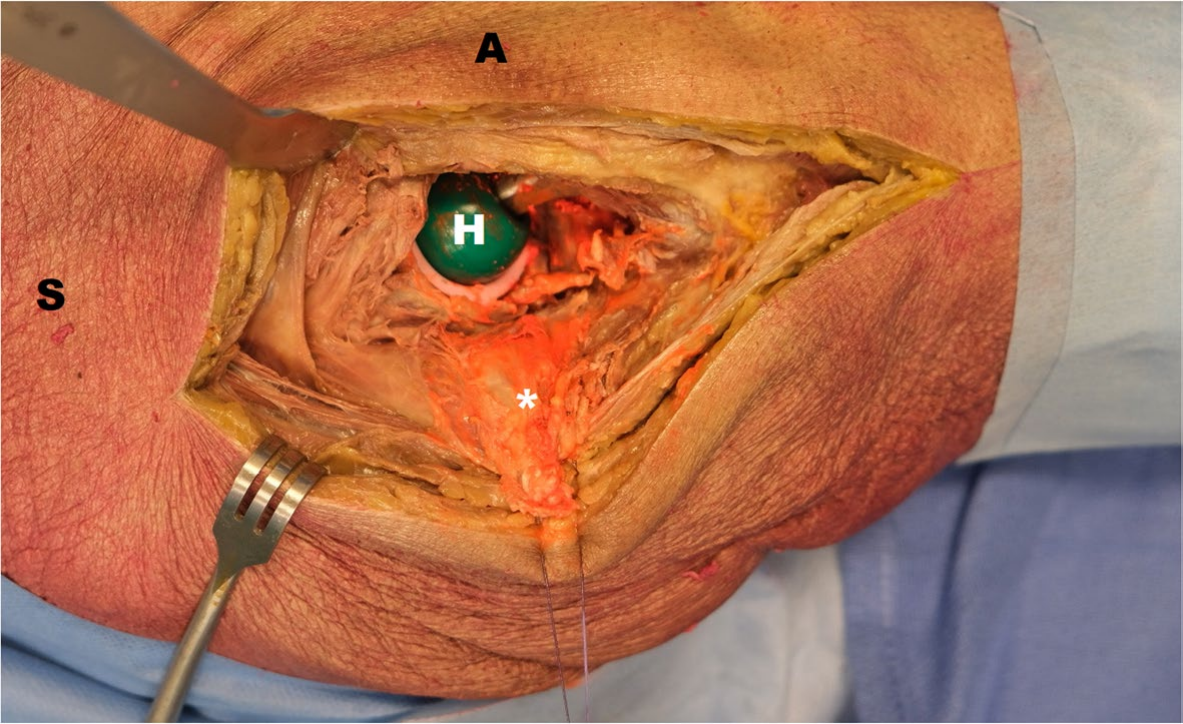
One-step exchange using a posterolateral approach. Retracting the external rotators and posterior capsule reveals the infected THA. S = superior, A = anterior, H = head of the implant, * = orange GloGerm powder mimicking the bacterial load

Second, a one-step exchange was simulated and carried out using all precautions one would normally take. The cadaver was placed in lateral decubitus using pelvic supports. The operating team consisted of a lead surgeon (SG), an assisting surgeon (JB), a scrub nurse (GV) and a circulating nurse (PD). After routine prepping (chlorhexidine/alcohol solution) and draping, a posterolateral approach was performed (Fig. 3). Debridement of clearly infected (orange) tissue was carried out from the start, followed by explantation of the prosthesis and subsequent pulsatile lavage with 6 L of saline. At this point randomization determined which clean phase protocol was followed, i.e. no, some or full additional measures (Fig. 2). Finally, a new prosthesis was implanted.

At the end of the experiment 9 one-step exchanges had been performed, with 3 levels of bacterial load (1, 2.5 and 5 cc of fluorescent power behind the cup, along the stem, and in the soft tissues) and using 3 different clean phase protocols.

### Variables and outcome measures

In order to determine the effect of different clean phase protocols on contamination of the sterile field the room was made pitch-dark and standardized UV light-enhanced photographs (Fujifilm X-T30 camera, focus 8, shutter speed 2, ISO 4000, diaphragm 3.5–4) were obtained of 1) the gloves of the operating team, 2) the instrument table, 3) the drapes, and 4) the wound. Blinded photographs were sent to the members of the Belgian Hip Society who were asked to rank them from most clean to most contaminated (www.survio.com – online survey tool). Thirty-eight out of 152 (28%) members responded.

In order to determine whether or not it is possible to re-implant the prosthesis completely clean, the implant was taken out again at the end of the one-step exchange and inspected for contamination under UV light.

### Statistical analysis

Friedman’s test was used to explore differences in ranking of cleanliness of the photographs of the nine experiments. In order to assess agreement among raters Kendall´s coefficient of concordance was used. If significant differences were found, post hoc testing was performed, again using Friedman´s test (Kendall´s W test), to explore differences between the 3 clean phase protocols. If significant differences were found, post hoc Wilcoxon signed rank testing was performed, to compare clean phase protocols amongst themselves. Given the presence of three groups, Bonferroni correction applied and therefore significance was set at *p* ≤ 0.017. Effect sizes (*r* = n cases / √Z) are furthermore reported.

## Results

### What is the effect of different clean phase protocols on the sterile field?

Statistical analysis (Table 1) showed that the differences in ranking of cleanliness (between the 9 experiments, but also between the 3 clean phase protocols) were significant (*p* < 0.000). Rater agreement ranged from moderate (0.360) for drapes to very good (0.808) for gloves. The gloves, the instrument table, the drapes and the wound were all considered significantly cleaner after a clean phase using full additional measures than after clean phases using partial or no additional measures (*p* < 0.000, medium to large effect sizes).

**Table 1:** Statistical analysis and comparison of the results of the three different clean phase protocols

	Friedman test (9 groups)	Kendall´s W test	Friedman test (3 clean phase protocols)	Kendall´s W test	Post hoc Wilcoxon signed rank testing				
					Most clean	↔ (significance) (effect size)	Intermediate clean	↔ (significance) (effect size)	Most contaminated
Gloves	*p* < 0.000	0.837	*p* < 0.000	0.808	Full	*p* < 0.000* *r* = 0.868	Partial	*p* < 0.000* *r* = 0.509	None
Table	*p* < 0.000	0.762	*p* < 0.000	0.661	Full	*p* < 0.000* *r* = 0.815	None	*p* = 0.935	Partial
Drapes	*p* < 0.000	0.422	*p* < 0.000	0.360	Full	*p* < 0.000* *r* = 0.786	Partial	*p* = 0.093	None
Wound	*p* < 0.000	0.810	*p* < 0.000	0.474	Full	*p* < 0.000* *r* = 0.632	Partial	*p* < 0.000* *r* = 0.502	None

^*^indicates statistical significance

**Fig. 4:**
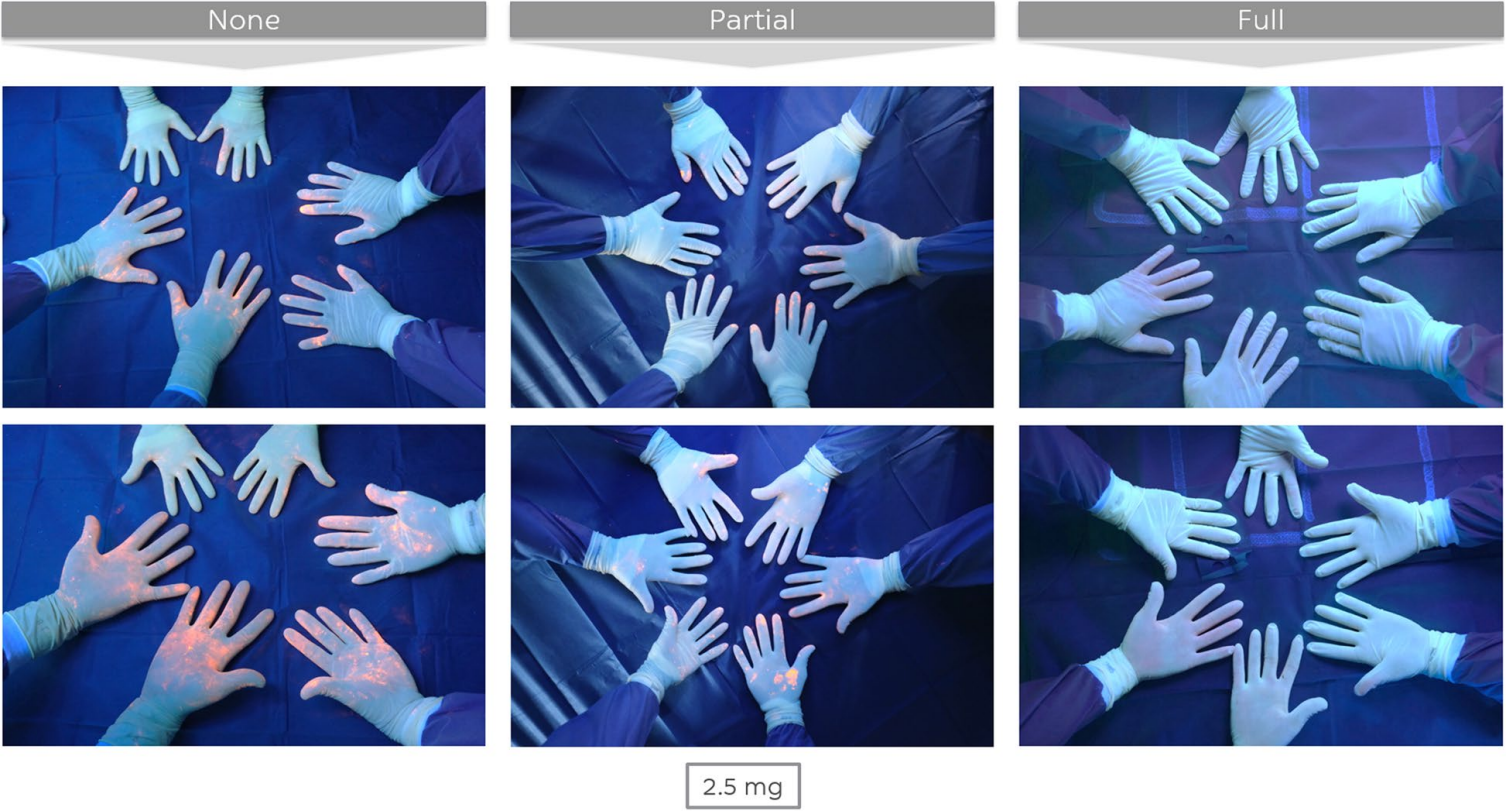
Gloves of the surgical team at the end of the one-step exchange for three different clean phase protocols (bacterial load of 3 × 2,5 mg GloGerm powder)

Changing gloves during the one-step exchange (clean phases with full and partial additional measures) led to cleaner gloves at the end of the procedure (Fig. 4). However, gloves remained significant cleaner (*p* < 0.000) during a one-step exchange using a clean phase with full additional measures, when compared to a clean phase with partial additional measures. Stated in a different way, fresh gloves will – for a degree – become contaminated again, if the sterile field is not completely renewed.

**Fig. 5:**
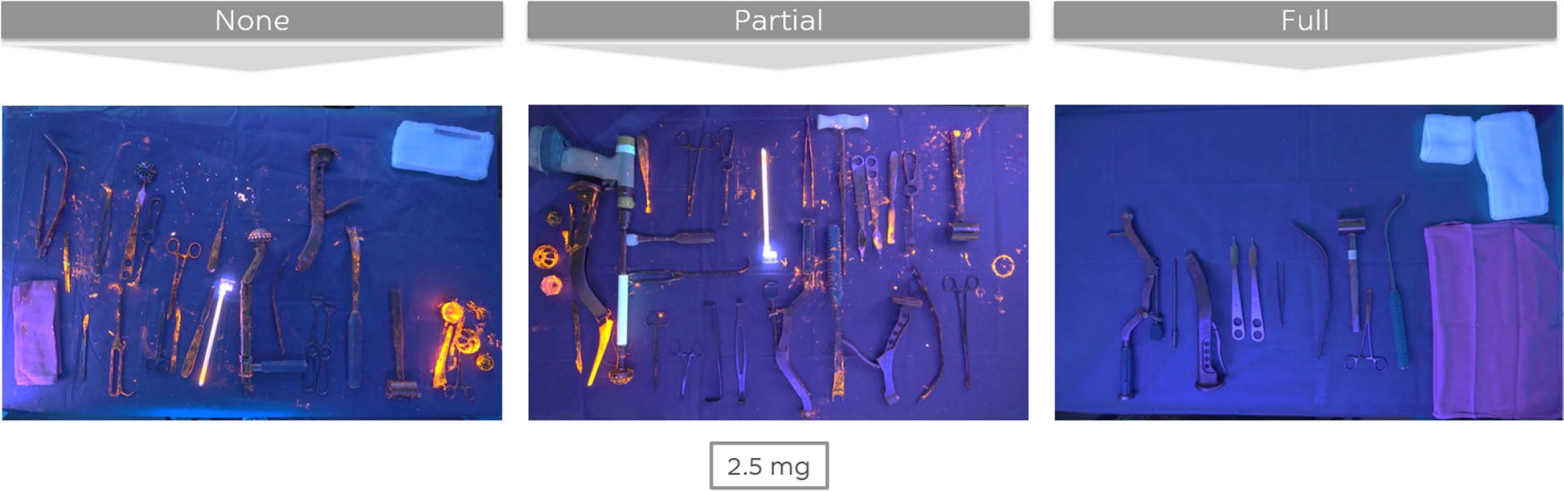
Remaining instrument table at the end of the one-step exchange for three different clean phase protocols (bacterial load 3 × 2,5 mg of GloGerm powder)

The instrument table (Fig. 5) at the end of a one-step exchange using a clean phase with full additional measures was rated as significantly cleaner than procedures making use of clean phases with partial of no additional measures. No significant differences were observed when comparing the latter two protocols amongst themselves. Stated in a different way, the contamination of the instrument table occurs during the dirty phase and partial clean phase measures do not seem to influence its contamination at the end of the procedure.

**Fig. 6:**
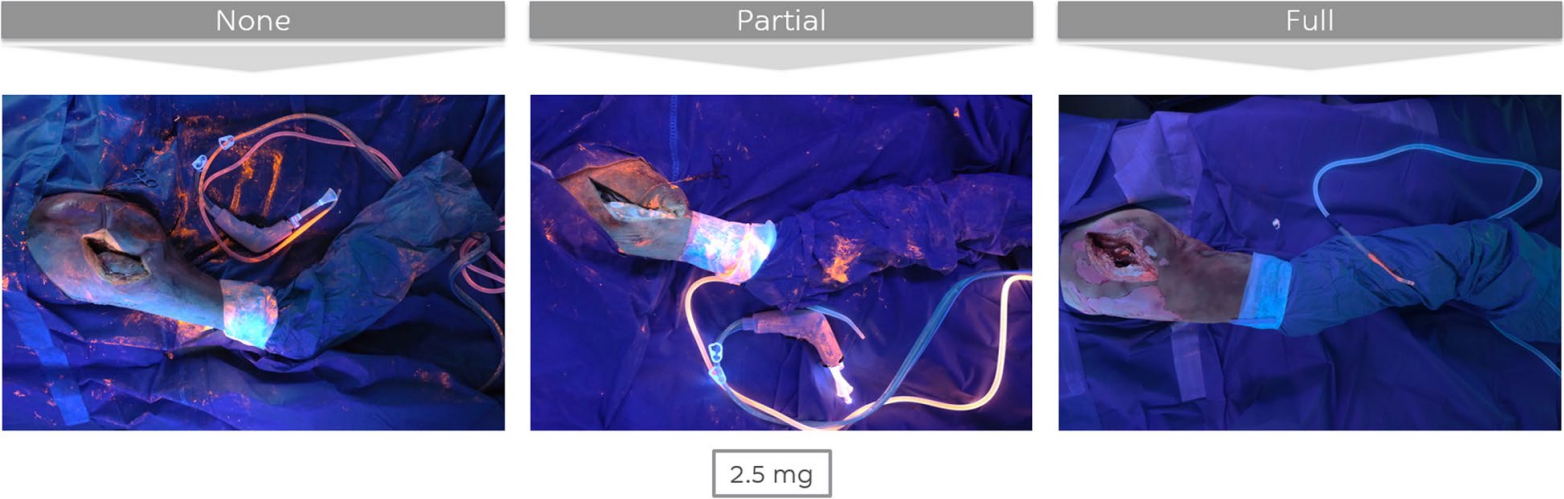
Drapes at the end of the one-step exchange for three different clean phase protocols (bacterial load 3 × 2,5 mg of GloGerm powder)

Similar observations were made for the drapes (Fig. 6). Drapes were most clean after a clean phase with full additional measures, while no significant differences were found when comparing the other two protocols. Stated in a different way, only changing the U-drape does not influence the perception of contamination of the drapes, while changing all drapes does. Of note, the stockinette was often the most contaminated drape.

**Fig. 7:**
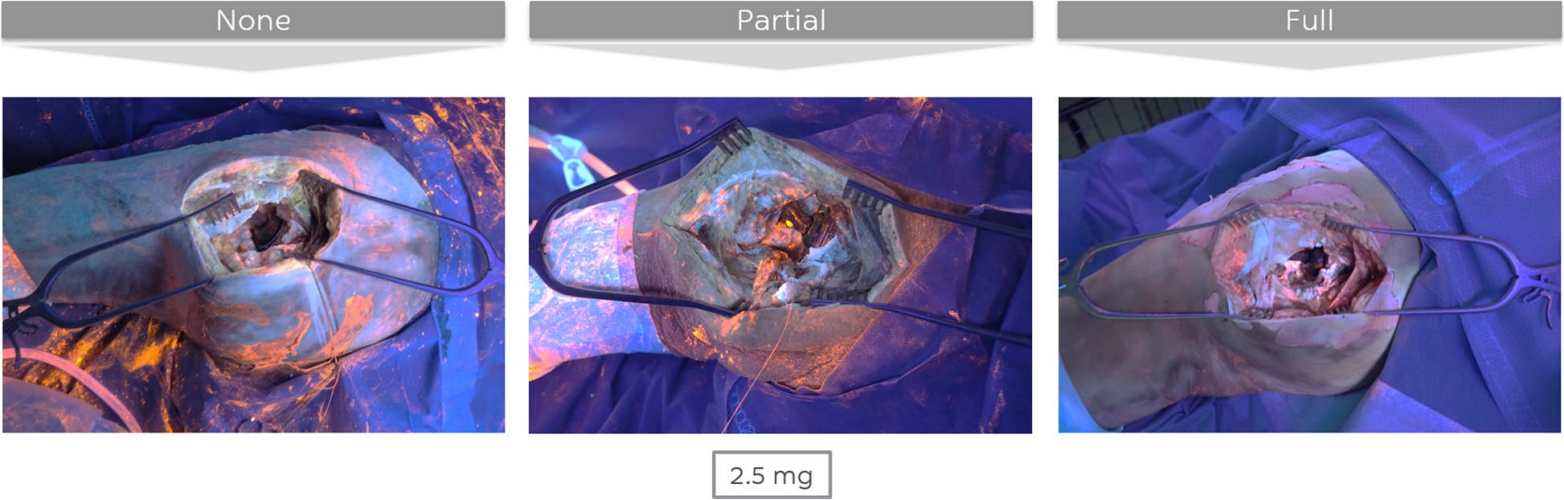
Surgical wound at the end of the one-step exchange for three different clean phase protocols (bacterial load 3 × 2,5 mg of GloGerm powder)

The wound (Fig. 7) after a clean phase with full additional measures was significantly cleaner than the wound after a clean phase with partial additional measures, which was in turn significantly cleaner than the wound after a clean phase with no additional measures.

### Is it possible to re-implant the prosthesis completely clean?

**Fig. 8:**
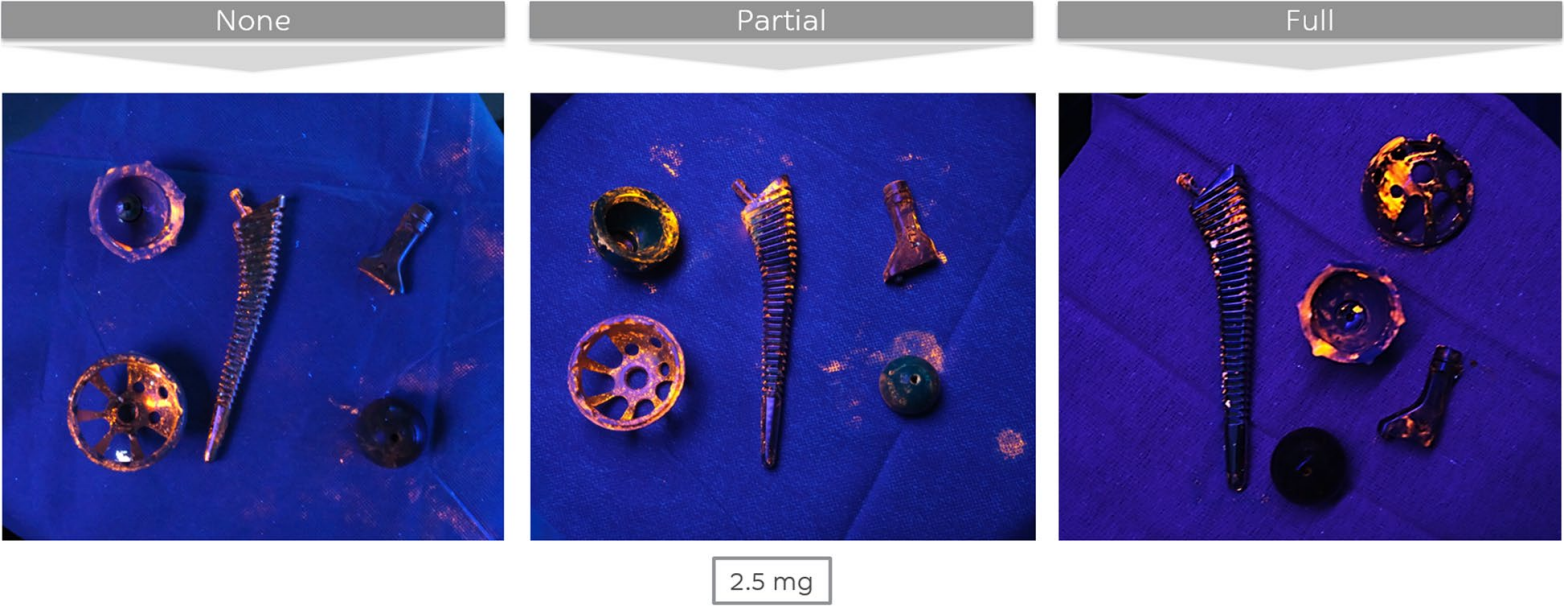
The newly implanted THA at the end of the one-step exchange for three different clean phase protocols (bacterial load 3 × 2,5 mg of GloGerm powder)

Without exceptions, all re-implanted joint replacements turned out to be contaminated with some orange GloGerm powder at the end of the one-step exchange regardless of the followed clean phase protocol or the amount of bacterial load at the beginning of the surgical procedure (Fig. 8). Therefore, in our experiments, it was not possible to re-implant the new prosthesis completely clean, even from a macroscopic point of view.

### Other relevant findings?

The extra costs associated with a partial and a full clean phase for our hospital and for a surgical team of three are € 10.86 and € 120.30, respectively (supplement 1).

## Discussion

Treatment of PJI remains challenging and it is essential to get the basics right. This cadaveric study therefore investigated the effect of different clean phase protocols on contamination of the sterile field during one-step exchanges of infected implants. Although it was shown that a more extensive clean phase helps to significantly reduce contamination of the sterile field before re-implantation, some contamination of the new implant appears inevitable.

This experimental cadaveric study has its limitations. Firstly, the absence of bleeding tissue might have influenced operating time, timing and number of surgical actions, and distribution of fluorescent powder around the wound and drapes. It is also possible that the fluorescent powder used is more adherent than bacteria and therefore more difficult to eradicate, which could have led to a visual overrepresentation of contamination. Secondly, there was no laminar air flow on the site of the experiment, although its relevance is questionable [14]. Thirdly, we used visual interpretation of the degree of contamination, rather than a quantitative evaluation. It must be stated that these ratings were done by experienced hip surgeons with generally high inter rater agreement and large effect sizes. Fourthly, one could argue that antiseptics would kill bacteria, and therefore not all contamination observed is still relevant. Lavage with antiseptics however is still controversial with opponents being concerned about cytotoxicity and bacterial rebound.

Finally, although it was illustrated that a full clean phase leads to the highest level of bacterial load reduction, definitive recommendations for clinical practice cannot be drawn from this experimental setup. Nevertheless, considering the difficulties associated with research in the field of septic surgery, performing a clinical RCT on this topic will be extremely difficult and in light of the results above, perhaps even unethical.

### What is the effect of different clean phase protocols on the sterile field?

It was illustrated clearly that the gloves, the instrument table, the drapes and the wound after a clean phase with full additional measures are significantly cleaner than those after a clean phase with partial or no additional measures. Partial measures were able to reduce some of the contamination of the gloves and the wound, but had no effect on the drapes and the instrument table. Although, the extra costs (€ 120.30) and extra operating time (approximately 10 min) spent on a full clean phase seem manageable, perhaps the biggest concern is the added layer of complexity to an already complex procedure. Many additional actions are required, which can cause disruption of sterility themselves and can lead to increased turbulence in the operating room. The extent of the clean phase therefore is a trade-off between theoretical bacterial load reduction and what is feasible in daily clinical practice. It is our opinion that in high-volume musculoskeletal infection centres with well-defined protocols and well-trained staff, a full clean phase should perhaps be preferred over a partial clean phase, as the benefits of the latter are limited. In centres where septic surgeries are only occasionally performed, some clean phase measures should still be possible without disrupting the surgical flow too much, e.g. providing a new U-drape and stockinette, changing the tip of the diathermy and suction, and the entire team changing gloves.

### Is it possible to re-implant the prosthesis completely clean?

In our experiments, we were unable to re-implant the new prosthesis entirely clean, even when minimal bacterial load was present from the beginning and a full clean phase was performed. This observation underlines the importance of adequate tissue levels of appropriate antibiotics at the time of implantation (which should therefore be given at induction and not withheld until cultures have been obtained) and might also provide a rationale for the use of antibiotic-loaded cemented implants during one-step exchange [15, 16].

The usage of fluorescent GloGerm powder allowed us to get instant feedback on our actions during a one-step exchange of the hip and therefore this set-up might also be used as a training tool for surgeons treating musculoskeletal infections. Several observations were of particular interest to us. While it is common practice for the lead surgeon to change gloves at certain stages during the operation, we noticed that the gloves of the scrub nurse were often the most contaminated. Another custom in centers where they perform a partial clean phase, is to provide a new U-drape. However, we noticed that the stockinette surrounding the leg is often equally or more contaminated. It would therefore make sense to change this as well since the leg is manipulated repeatedly during the clean phase for exposure and testing of stability and leg length.

Finally, although inspection of the explanted prosthesis might reveal useful information on sizes, wear, and impingement, it should be regarded as the most contaminated object of the entire procedure and therefore handled accordingly.

## Conclusion

This study investigated the effect of different clean phase protocols on contamination of the sterile field during one-step exchanges of infected THAs on cadavers. It was found that a more extensive clean phase helps to significantly reduce contamination before re-implantation. We acknowledge that some contamination of the new implant appears inevitable and that the extent of a clean phase therefore is a trade-off between theoretical load reduction and what is feasible in daily clinical practice. However, we advise surgical teams in high volume musculoskeletal infection centres to try and incorporate a clean phase with full additional measures into their work flow, as partial measures seem to be a poor compromise. A comprehensive registry on septic surgeries could help to better understand the clinical relevance of a clean phase. 

## Supplementary Information

**Additional file 1.** Supplement 1.

## Data Availability

Upon request.
