# Supplementary material for: On the value and limitations of incorporating a “clean phase” into the surgical treatment of prosthetic joint infections – an illustrative cadaveric study using fluorescent powder
Source: J Exp Orthop. 2022 Mar 21;9:28. doi: 10.1186/s40634-022-00467-x (PMC8938588; doi:10.1186/s40634-022-00467-x)
Supplement: Supplementary file 1 — Additional file 1. Supplement 1. [file 40634_2022_467_MOESM1_ESM.docx]

**Supplement 1**

|  | **Partial clean phase** | **Full clean phase** |
| --- | --- | --- |
| U-drape | € 5.39 | € 5.39 |
| Gloves x 3 | € 4.80 | € 4.80 |
| Suction tip | € 0.67 | € 0.67 |
| Suction hose |  | € 2.05 |
| Gowns x 3 |  | € 9.45 |
| Chlorhexidine/alcohol solution |  | € 1.94 |
| Instrument tray |  | € 34.0 |
| Drape kit for posterolateral |  | € 62.0 |
| Total | € 10.86 | € 120.30 |

In the cadaveric experiment no electric cautery was used – this would add another € 8.42 and € 19.36 to the partial (cautery tip) and full clean phases (completely new electric cautery with fume exhaust unit), respectively
